# The Metropolitan Hospital Sunday Fund

**Published:** 1902-12-13

**Authors:** 


### THE METROPOLITAN HOSPITAL SUNDAY FUND.

### A RECORD YEAR.

At the annual general meeting of the Metropolitan Hospital Sunday Fund, held on Tuesday (9th inst.) at the Mansion House, the chair was taken by the Lord Mayor, Sir Marcus Samuel. He was supported by the Bishop of London, Earl Stamford, and Sir Edmund Hay Currie, secretary of the Fund. Letters of regret had been received from several supporters who were unable to be present. Among these were Sir Sidney H. Waterlow, Vice-President, the Bishop of Stepney, the Archdeacon of London, the Rev. M. R. Neligan, Mr. R. B. Martin, Mr. Alfred Fripp, and others. There was, however, a good attendance, many of those whose excellent work in connection with the Fund is well known being present.

### Not Satisfied Yet.

The first business was to receive and approve the report for 1902, and this was proposed by the Bishop of London, who congratulated the new secretary (Sir Edmund Hay Currie) on the fact that his first year of office had been marked by such a splendid success, the contributions having reached the highest total yet known, *i.e.* £62,669 12s. 7d. It must be fully realised, his Lordship continued, that the existence of other hospital funds (such as King Edward's Fund) did not do away with the necessity for the Metropolitan Hospital Sunday Fund. He thought, indeed, that it would be found in actual working out that King Edward's Fund contributed chiefly towards hospital extension, and the Sunday Fund towards maintenance. He looked forward to seeing the yearly contributions raised to £100,000 in the near future. Those who knew most about the hospitals realised that the need for this and kindred funds was just as great as ever. "Don't let us rest upon our laurels," he concluded, "but go forward. This Fund draws us all together; all denominations are united in one effort to help the poor and suffering in London."

Canon Fleming, in seconding, said everyone present would endorse the Bishop's aspiration that £100,000 should be raised: it was a sum for which they had long been striving. Medical science, skill, and nursing, he thought, had done all that could be done at present towards the healing of disease, but it remained for the benevolent to help a fund which brought the benefits of science within the reach of the poor. He rejoiced to see that 65 new places of worship had been added to the number of those giving an annual collection; and even more than a large increase in the sum of money subscribed, he would desire to see that all congregations of all denominations should be brought into the number. The report was adopted.

### From a Business Point of View.

The next resolution was proposed by the Bishop of Barking: "That the council for the year 1902 be re-elected for the year 1903, with the Right Hon. Lord Sandhurst, G.C.S.I., G.C.I.E.; the Right Hon. Sir Joseph Dimsdale Bart., P.G., M.P., K.C.V.O.; Charles Ernest Tritton, Esq., M.P., Sir Edward Durning Lawrence, and the Rev. Brook-Deedes, to fill vacancies. The proposer thought it would be taken for granted that those who had seen the Fund through its most successful year should be asked to continue in office.

The seconder was the Rev. R. L. Allwork, Vicar of St. Saviour, Walthamstow, who said he looked upon this Fund as the one that gave the best return for one's money. His congregation was unable to afford more than £10, while at the district church attached not more than £3 could be collected; nevertheless, something like a benefit of £45 was received. The council, as proposed, was elected.

### Why the Coronation Collections were a Record.

The third resolution—"That the best thanks of the meeting be given to George Herring, Esq., for his generosity, which has assisted the Fund to secure the record amount of £62,669"—was proposed by Sir Henry Burdett, who said that the name of Mr. George Herring would always stand out prominently as that of one who had made this record year possible. He had come forward for three consecutive years with the munificent gift of £10,000 to this Fund. He (the speaker) had always said that when a man who had made his fortune commenced to give largely to approved objects of charity he began to realise the blessedness of living rather than existing. He thought Mr. Herring had already begun to breathe something of that higher atmosphere. This year he had altered the form of his gift, and had enabled clergymen and ministers to say to their congregations that every £1 they gave on Hospital Sunday would have 5s. added to it by Mr. Herring's generosity.

This development of Mr. Herring's gift proved that the giver had put his head as well as his heart into what he was doing on Hospital Sunday. In so acting Mr. Herring, by his gift of £11,500, had secured a new revenue for the hospitals of £10,000 a year. That is to say the amount contributed by the whole of the congregations, which had stood at a total of about £36,000 for several years, was this year raised by Mr. Herring's intelligent benevolence to £46,000—a splendid result. Foreigners often wondered how the English maintained the largest buildings in their great cities by voluntary contributions. The secret lay in acts like that of Mr. George Herring, who not only gave his money, but also his time and thoughts to the furtherance of great charitable movements like Hospital Sunday. Besides Mr. Herring had for years devoted much time and energy to the up-keep and management of the North-West London Hospital, in which he took the deepest interest. Sir Henry claimed that it was good for the community to emphasise the practical and fruitful benevolence of a munificent giver such as Mr. George Herring, who he had little doubt had experienced very genuine gratification that the Hospital Sunday collections of the Coronation year, largely through his foresight and encouragement, would constitute a record. Every resident in London owed him grateful thanks, and Sir Henry hoped that Mr. Herring might realise this very real and genuine feeling of the people towards himself.

The Earl of Stamford, in seconding, said there was a great opening for millionaires. If one would step forward and follow Mr. Herring's example, the total of £100,000 would soon be reached.

The resolution was carried by acclamation.

### A Suggestion.

The 14th of June was fixed for Hospital Sunday of 1903, and the cordial co-operation of all ministers of religion within the metropolitan area was invited in the usual way in a resolution proposed by the Chief Rabbi and seconded by the Rev. Prebendary Ridgway. The former thought it would be advisable, in the week preceding Hospital Sunday, to organise a Mansion House meeting with a special speaker whose oratory would stir up a greater amount of interest. The latter said two causes contributed to the increased contributions of his congregation, *i.e.* the peculiar form of Mr. Herring's gift (the most important part, in his opinion) and the letter sent out by the Lord Mayor, which reached many who did not hear the appeals of the clergy.

Mr. James Gamble thought the contributions from the

City churches were miserably inadequate, and a gentleman from the audience wanted to be assured that none of the Hospital Sunday money would go to the medical schools. The assurance was given.

Change in the Basis of Award.

Mr. F. H. Norman proposed an alteration in the laws of the constitution, the effect of which was to deduct expenditure on buildings from the basis of award.

Sir William Church seconded, and the alteration was accepted.

In replying to a vote of thanks, the Lord Mayor said the suggestion of the Chief Rabbi with regard to a special meeting at the Mansion House before the date fixed for the collections had his warm approval. He would also gladly sign a circular letter, or in any other way forward the interests of the Fund.

